# Neurovascular dysfunction in the development and progression of neuroinflammatory diseases

**DOI:** 10.3389/fncel.2026.1741928

**Published:** 2026-03-13

**Authors:** Jamila Gowdy, Julie Ahn, Robert H. Miller, Yusra Islam

**Affiliations:** Robert H. Miller Laboratory, Department of Anatomy and Cell Biology, George Washington University, Washington, DC, United States

**Keywords:** B cells, blood–brain barrier, endothelial dysfunction, glial cells, multiple sclerosis, neurodegeneration, neuroinflammation, neurovascular unit

## Abstract

The neurovascular unit (NVU) is critical for brain homeostasis through its roles in maintenance of an effective blood brain barrier (BBB) and regulation of cerebral blood flow. Perturbation of the NVU is a hallmark of the pathology of multiple neurodegenerative diseases resulting in loss of BBB integrity, neuroinflammation and neuronal dysfunction. The NVU is a complex structure composed of endothelial cells, pericytes, as well as central nervous system (CNS) glial and neuronal components. While the importance of the CNS vasculature in health and disease is well established, the mechanisms underlying vascular pathology and its contributions to neurodegenerative diseases are less well defined. Neuroinflammation and reactive gliosis occurs in the majority of neurodegenerative diseases and recent studies suggest that immune mediated disruption of the BBB contributes to the induction of reactive gliosis and neuronal dysfunction. Potential consequences of NVU disruption include immune-driven vascular inflammation and leukocyte infiltration in Multiple Sclerosis (MS), protease-mediated tight junction degradation in ischemic stroke (IS), *α*-synuclein–associated endothelial dysfunction in Parkinson’s Disease (PD), amyloid-*β*– and tau-induced pericyte injury in Alzheimer’s Disease (AD), and complement-mediated vascular damage in Amyotrophic Lateral Sclerosis (ALS). Here we review the nature of NVU perturbations in these common neurodegenerative diseases, with an emphasis on the contribution of immune modulation of BBB disruption in neuropathology and disease progression.

## Introduction

Diseases of the mature central nervous system (CNS), including Parkinson’s disease (PD), Alzheimer’s disease (AD), Multiple Sclerosis (MS), Ischemic Stroke (IS), and Amyotrophic Lateral Sclerosis (ALS), have distinct characteristics including the populations of cells affected, the molecular basis of the pathology, and the location of the disease. While distinct diseases, they share some common characteristics that include the activation of an immunological response and the perturbation of the functions of the neurovascular unit (NVU).

**Figure 1:**
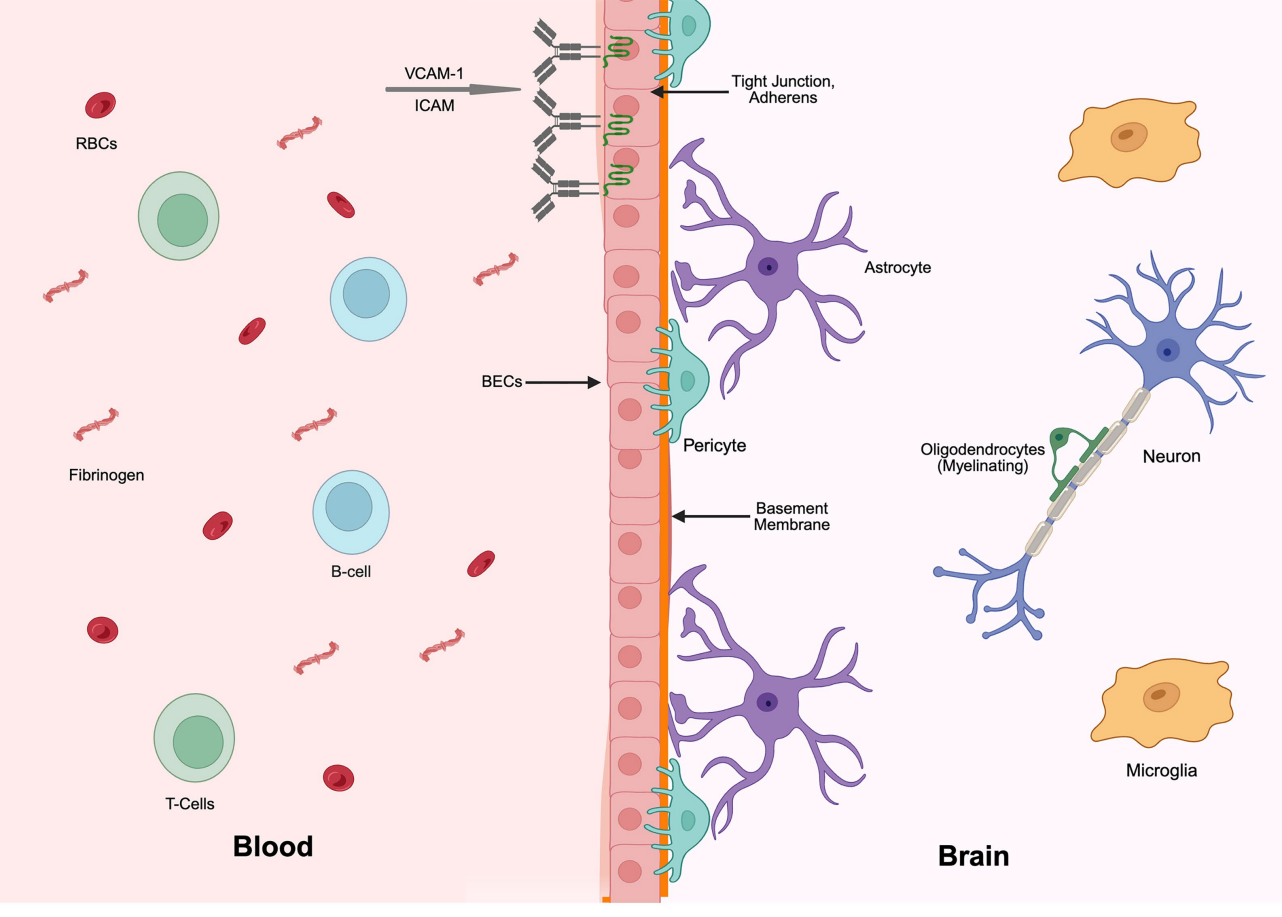
Schematic of a healthy NVU consisting of astrocytes, oligodendrocytes, microglia, pericytes, and brain endothelial cells (BECs). The blood–brain barrier (BBB) comprises BECs held together by tight junctions and claudins. Pericytes and the basement membrane support the BBB and limits the flow of chemicals from the blood into the brain. Astrocyte endfeet wrap blood vessels to help reinforce the integrity of the NVU while simultaneously supporting neuron signaling and oligodendrocyte metabolism. Resting microglia serve as resident macrophages of the CNS helping to clear out debris. Red blood cells (RBCs) and immune cells, including leukocytes and B-cells, circulate in the blood. This figure was generated using BioRender.

The NVU is a complex, dynamic structure composed of multiple different cell types (Figure 1). While the NVU was originally thought to simply be the basis of the blood brain barrier (BBB), recent studies indicate it is a far more dynamic element of the CNS (Badaut et al., 2024). The BBB acts as a selective barrier to prevent the unrestrictive passage of peripheral immune cells, pathogens and large molecular moieties from blood to the parenchyma of the brain (Friedman et al., 2025). The BBB is not a passive barrier but facilitates the active transport of selective substances between the lumen of the vasculature and the parenchyma of the brain (Erickson and Banks, 2018). Classical studies using dye tracing at the ultrastructural level (Reese and Karnovsky, 1967) demonstrated that the physical location of the barrier was at the interface between the endothelial cells lining vessels of the brain. These cells, commonly referred to as Brain Endothelial Cells (BECs), have been the subject of extensive investigation, and much is known regarding their molecular composition and response to injury (Yuan et al., 2023). Mature BECs are interconnected by two types of junctions: adherens junctions that provide structural support and maintain the close apposition of cells, and tight junctions, which are formed through interactions among membrane-associated proteins, including cytoplasmic zonula occludin proteins (e.g., ZO-1), transmembrane claudins (most notably claudin-5), and tight-junction–associated MARVEL proteins (TAMPs) such as occludin and tricellulin (Hudson and Campbell, 2021; Raleigh et al., 2010). Tight junctions fuse the plasma membranes of adjacent cells and are the critical element in the generation of the barrier. Considerable evidence suggests that BECs both respond to neuropathological triggers and influence the progression of the disease through the release of a variety of signaling molecules and cytokines (Sá-Pereira et al., 2012; Krueger and Bechmann, 2010; Bhattacharya et al., 2020).

The formation of the BBB by BECs is regulated by interactions with other cells within the NVU, including pericytes and astrocytes. Pericytes extend processes along blood vessels and are located between the endothelial cells and astrocyte endfeet (Sá-Pereira et al., 2012; Krueger and Bechmann, 2010; Bhattacharya et al., 2020). Based on cytoskeletal features and their exact positioning along the vascular tree, pericytes can be categorized into ensheathing pericytes (arteriole–capillary junction), mesh pericytes (pre- and post-capillary venules), and thin-strand pericytes (mid-capillary segments; Brown, 2019). Although these classes have been morphologically identified, brain pericytes do not appear to exhibit distinct subtypes at the transcriptomic level and collectively appear to be critical for the formation and maintenance of the BBB (Blanchette and Daneman, 2015; Dalkara et al., 2011; Vanlandewijck, 2018). One of the major signaling pathways between BECs and pericytes is through the PDGFb/PDGFbR pathway, and inhibition of this pathway results in failure of CNS vessel formation and stabilization (Daneman, 2010; Armulik et al., 2010; Betsholtz and Keller, 2014). Other signaling pathways such as Notch and TGF-β have also been shown to be important in the adhesion, proliferation, and migration of pericytes (Krueger and Bechmann, 2010), and their perturbation results in a breakdown of the BBB, hemorrhaging, and perivascular edema (Bhattacharya et al., 2020; Allinson et al., 2012; Gaengel, 2009). Given their critical role in the maintenance of the NVU function, it is not surprising that pericytes have been implicated in a variety of neuropathological conditions.

Another crucial cellular element of the NVU is astrocytes (Liu et al., 2020; Aharoni et al., 2021; McConnell and Mishra, 2022). Astrocytes, which are the most abundant glial cell type in the CNS, are a major class of spatially and functionally heterogeneous glial cells (Bocchi et al., 2025; Serrano-Pozo et al., 2024; Miedema, 2024; Schitine et al., 2015; Bacchi, 2025; Qian et al., 2023) that support neuronal metabolism, regulate synaptic function and myelination, and respond to CNS injury (Alberini, 2018; Babenko et al., 2024; Barres, 2008; Ishibashi et al., 2006; Sofroniew and Vinters, 2009; Stogsdill et al., 2023). Astrocyte processes cover the surface of most CNS vessels and are thought to play multiple roles, including formation and maintenance of the BBB. For example, under specific conditions, astrocytes release pro-inflammatory and anti-inflammatory cytokines that modulate BBB permeability (Manu et al., 2023; Michinaga and Koyama, 2019), while the retraction of astrocyte endfeet around CNS capillaries during inflammation increases BBB vulnerability and, potentially, CNS damage (Manu et al., 2023; Ponath et al., 2018; Salles, 2022).

In pathogenic states, perturbation of the NVU may result in hypoxia, increased inflammatory activity (Stanimirovic and Friedman, 2012), and a secondary cascade of events resulting in the disruption of the BBB (Balasa, 2021), in turn allowing the trafficking of proteins such as fibrin into the CNS. In diseases such as multiple sclerosis, active lesions are characterized by increased leukocyte infiltration (Sweeney et al., 2019) and lesions develop around small, inflamed veins (Gaitán et al., 2011), suggesting that damage to the NVU contributes to the development of neuroinflammatory diseases in the CNS. It is becoming increasingly evident that the pathology of many neurological diseases, regardless of the initial insult, reflects engagement of the immune system and NVU dysfunction. In this review, we integrate emerging evidence that immune–NVU interactions, particularly those involving B cells and glial populations, play a central role in BBB engagement across multiple neurodegenerative diseases, offering a unifying framework for disease progression that extends beyond disease-specific models.

## Multiple sclerosis

Multiple sclerosis (MS) is a chronic autoimmune, neuroinflammatory, demyelinating disease of the CNS. MS is characterized by localized myelin damage and axonal injury, with lesions predominantly located in brain and spinal cord. CNS white matter is comprised of myelinated and unmyelinated axons, astrocytes, oligodendrocytes, and vascular elements, with the high lipid content of myelin contributing to its opaque white appearance (Walhovd et al., 2014). Symptom onset in MS typically presents in young adults, with a higher prevalence in women than men (Liu et al., 2022), and includes motor dysfunction, tremors, fatigue, nystagmus, paralysis, ataxia, and vision impairment (Ortiz, 2014).

MS is a heterogeneous disease classified into relapsing–remitting MS (RRMS), secondary-progressive MS (SPMS), primary-progressive MS (PPMS), and progressive-relapsing MS (PRMS), with the most common type being RRMS, impacting people in early adulthood (Liu et al., 2022). In RRMS, patients experience relapses that temporarily dampen specific neurological functions, followed by a period of remission in which some functionality is regained. In some patients, the disease transitions to a more progressive SPMS condition (Cree and Hartung, 2025). The rate of progression is variable; some patients never transition to SPMS, while those who do, develop a steadily progressing form of the disease. By contrast, PPMS is characterized by continuous disease progression from onset. In comparison, PRMS is characterized by a constant disease progression that is accompanied by acute relapses with no remission (Dutta and Trapp, 2014).

The ambiguous nature of MS pathology makes reaching a definitive diagnosis difficult in earlier stages of the disease. Initial diagnoses based on changes in inflammation across different regions of the CNS was later amended to include measurements of oligoclonal immunoglobulin G (IgG) bands (OCB) in the patient’s cerebrospinal fluid (CSF; McDonald, 2001; Thompson et al., 2018; Deisenhammer et al., 2019). The increase in IgG levels, coupled with disease progression, likely reflects the differentiation of B cells into plasma cells in response to signals received from T helper cells (Th17; Vaillant, 2023; Graner et al., 2020).

**Figure 2:**
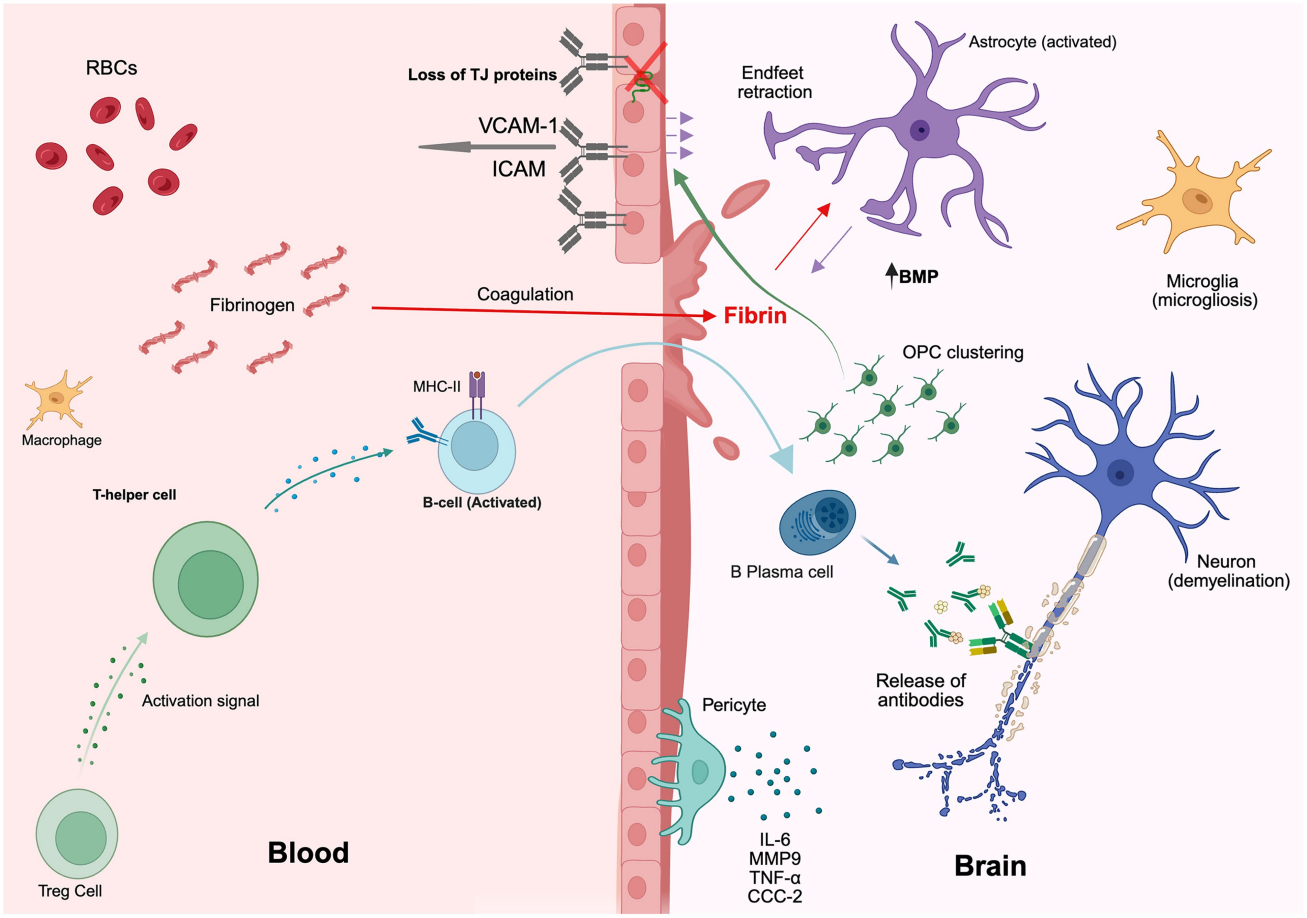
Representation of immunological involvement and dysfunction of the NVU in multiple sclerosis (MS). The loss of claudins and tight junctions, followed by astrocytic endfeet retraction, results in the disruption of the BBB. Immune cell transmigration is facilitated by an increase in the expression of VCAM-1 and ICAM on endothelial cells. Fibrinogen leakage into the brain initiates coagulation, while fibrin deposition promotes OPC clustering, which activates BMP signaling and inhibits oligodendrocyte differentiation. This blockade of differentiation drives OPCs to target astrocytic endfeet, preventing vascular ensheathment and contributing to their retraction. Local inflammation is a result of the weakened BBB, allowing the infiltration of macrophages, activated microglia (microgliosis), and plasma B cells in peripheral blood, T-regulatory cells (Treg) send activation signals, prompting T-helper cells to secrete IL-17, IL-21, and IL-22 as they shift into a pro-inflammatory state. This stimulates peripheral B-cells to shift to antibody-producing plasma B cells through the expression of major-histocompatibility complex II (MHC-II). The release of IL-6, MMP9, TNF-a, and CCC-2 from pericytes further amplifies neuroinflammation and the disruption of the BBB. This figure was generated using BioRender.

Lesion formation and local demyelination are associated with local inflammatory responses associated with infiltrating autoreactive immune cells (Cui et al., 2020; Cashion et al., 2023). At the level of the NVU, early signs of MS involve cytokine cascades triggered by T-cells, leading to the destruction of cerebral blood vessel integrity enabling the admission of inflammatory leukocytes into the CNS (Ortiz, 2014). One hypothesis is that a breakdown of tolerance established during the gestational and postnatal periods stimulates regulatory T cells (Tregs) to exhibit a pro-inflammatory state and secrete proinflammatory proteins (Cui et al., 2020; Tanaka et al., 2014). Consequently, cells such as peripherally activated microglia, activated B cells, and Th17 cells, are recruited into the CNS, resulting in either protective or pathogenic responses (Cashion et al., 2023; Tanaka et al., 2014). While the role of major classes of CNS cells in MS pathology has become more defined, the role of the NVU is often overlooked (Figure 2).

### Engagement of cells of the NVU in MS pathology

In MS, lesions typically form around blood vessels (Gaitán et al., 2011), where local demyelination of axons compromises their conduction potential, resulting in neurological deficits (Lassmann, 2003). Neurovascular cells, including BECs, pericytes, astrocytes, and microglia, are impacted during the development of MS pathology and may contribute to a pro-inflammatory state of the CNS (Daigle et al., 2025; Iadecola, 2017; Schreiner et al., 2022). Linkage between adjacent BECs weakens during inflammation as tight junctions are lost, allowing increased leukocyte trafficking into the CNS (Cayrol, 2008). Key molecules involved include VCAM-1, ICAM-1, IL-4, IL-10, TNFα, and IFN-*γ* (Cayrol, 2008; Raine, 1995; Cashion et al., 2023). Inflammatory cues also drive mislocalization of chemokines like CXCL12 and upregulation of leukocyte adhesion molecules on BEC surfaces, including VCAM-1 (Allavena et al., 2010), DARC (Minten et al., 2014), and ALCAM (Cashion et al., 2023; Cayrol, 2008). Notably, ALCAM knockout mice develop more severe experimental autoimmune encephalomyelitis (EAE), a widely used animal model for MS (Constantinescu et al., 2011), suggesting a compensatory role in maintaining BBB integrity (Lécuyer et al., 2017). These changes likely reflect BEC responses to inflammatory signals from surrounding cells, such as pericyte-mediated induction of adhesion molecules (Bisht et al., 2021).

During inflammatory episodes in the CNS, pericytes appear to induce constriction of cerebral blood vessels for extended periods (Fortune, 2025). Studies in animal and human tissue identified PDGFRb, CD13, *α*-smooth muscle actin (αSMA), NG2, and Atp13a5 as pericyte-specific markers (Kang, 2020; Guo et al., 2024), and in disease, an increase particularly in aSMA and NG2 expression usually indicates pericyte activation (Ferrara et al., 2016; Verbeek et al., 1994). It is important to note that while markers like PDGFRβ and NG2 are widely used, their specificity remains an active area of research. Markers such as αSMA are also expressed by vascular smooth muscle cells, and others, including PDGFRβ, are found on CNS fibroblasts and other mesenchymal cells, particularly near the meninges or in areas of injury (Yao, 2022). This cellular heterogeneity and marker overlap makes precise identification of the pericyte population challenging in pathological settings. In addition to reducing blood flow, pericytes facilitate the migration of immune cells into the CNS via the antigen-presenting molecule, MHC-II, promoting the recruitment of activated T-cells (Silva et al., 2021). Inflammatory T-cells appear to depend on MHC-II-presenting pericytes to proliferate as pericyte removal results in a decrease in the inflammatory T-cell population within the CNS (Silva et al., 2021), supporting the idea that pericyte dysfunction plays a role in MS and permits the transit of inflammatory cells across the BBB. For example, the loss of pericytes is associated with a reduction in tight junctions, decreased claudin-5 expression, and enhanced infiltration of blood-derived proteins into the CNS (Aharoni et al., 2021; Monsour and Borlongan, 2023), while activated pericytes may promote leucocyte adhesion (Choe, 2022) and secrete pro-inflammatory cytokines including TNF-*α*, IL-6, matrix metalloproteinase 9 (MMP9), and the chemokine CCL-2 (Bhattacharya et al., 2020; Cashion et al., 2023; Meyer-Arndt et al., 2023). The functions of MMP9 in the setting of neuroinflammation are complex, and it has been suggested that MMP9 may disrupt the vascular basement membrane, although it may also be that MMP9 overexpression results in pericyte loss and BBB hyperpermeability through compromising tight junctions (Dohgu et al., 2019).

**Figure 3:**
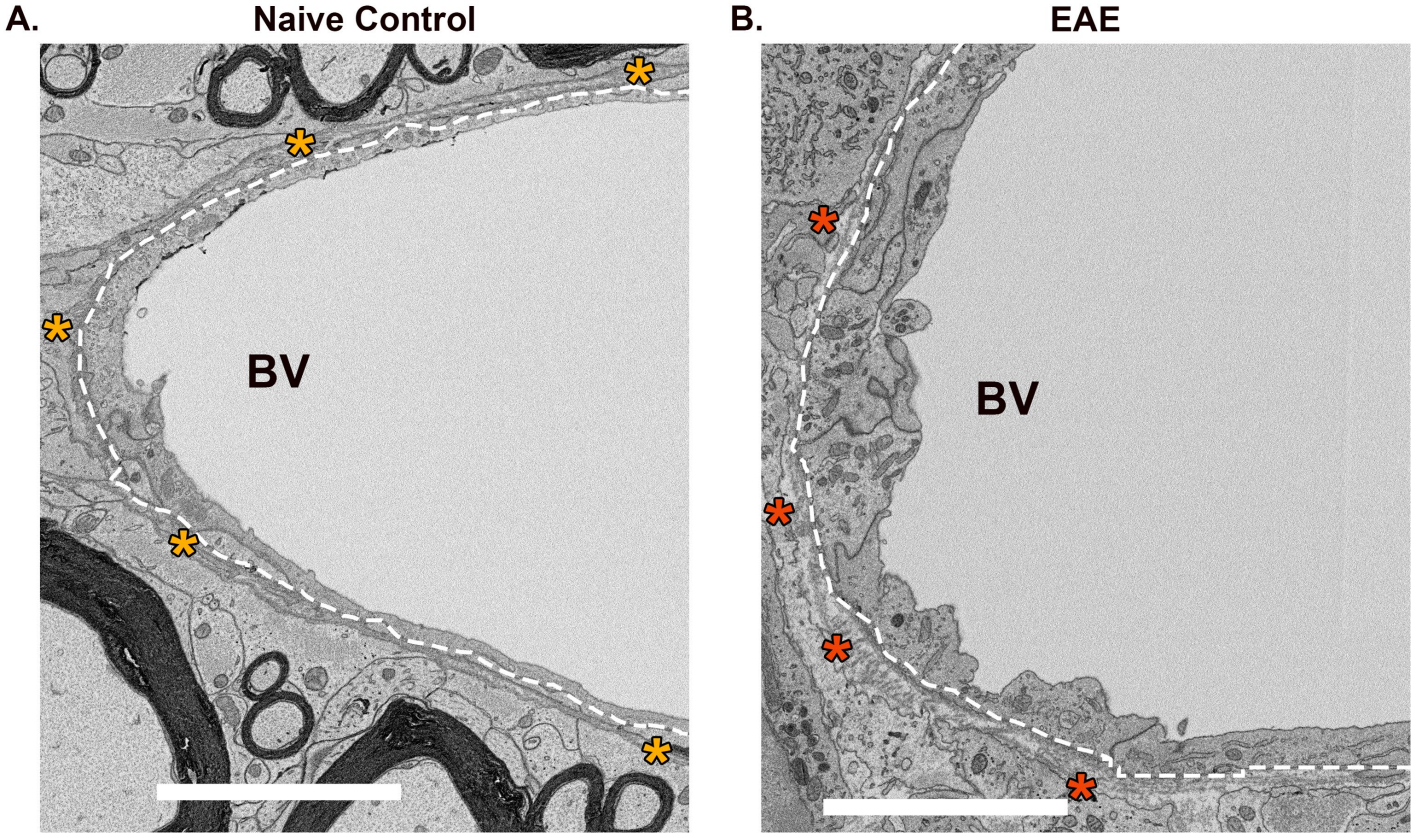
The NVU is altered in EAE, a mouse model of MS. Electron microscopy images of the NVU in **(A)** naïve control or **(B)** EAE mice. Endothelial cell lining the blood vessel is represented by the white dashed line. Yellow or red asterisks represent healthy or missing astrocyte endfeet, respectively. BV – blood vessel. Scale bars 5uM.

**Figure 4:**
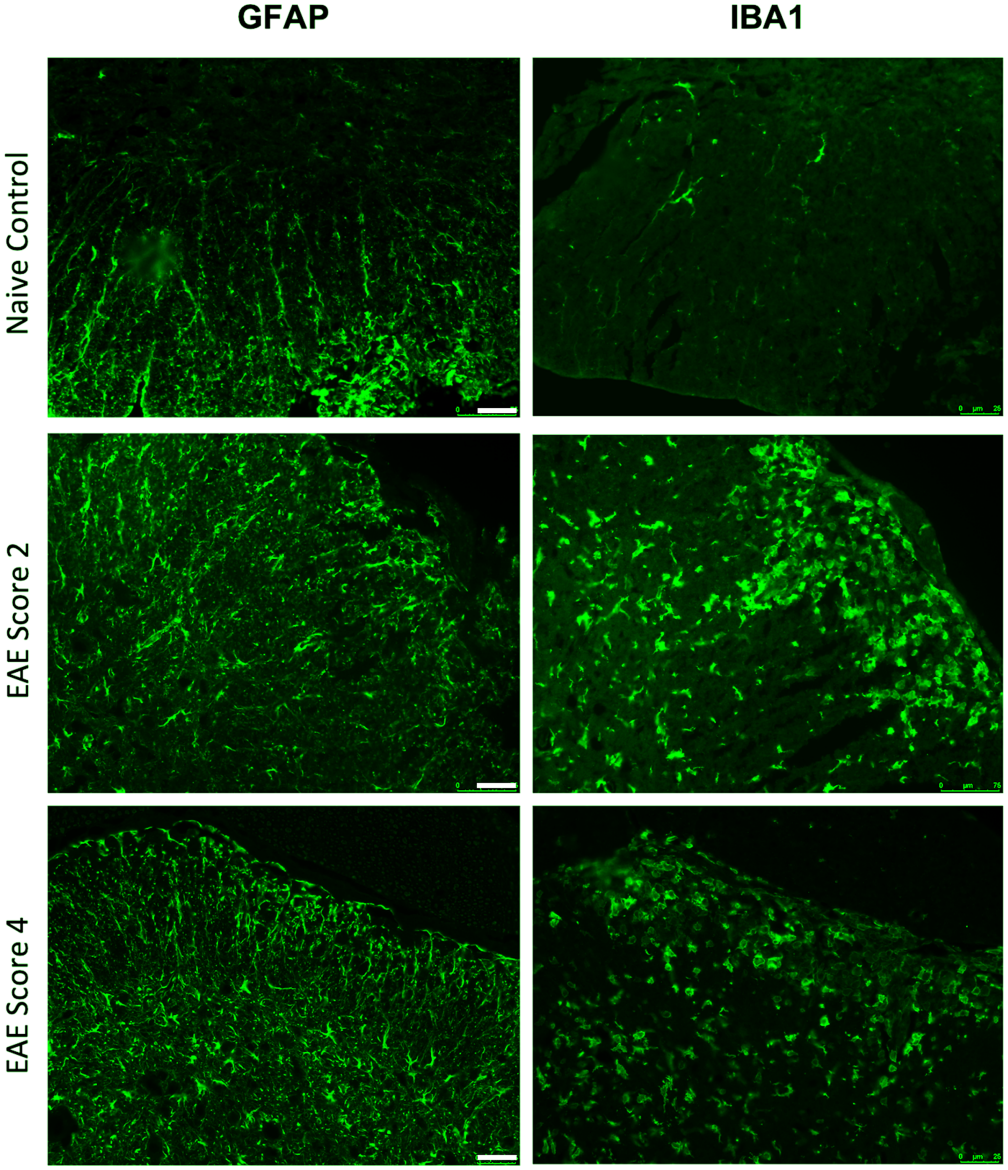
Astrocyte and microglial density increase with EAE disease severity. Representative IHC showing GFAP+ astrocytes and IBA1 + microglia/macrophages in spinal cords from naïve mice and EAE mice with clinical scores of 2 and 4. Scale bars 50uM.

Astrocytes are a central element in the pathology of MS. The retraction of their endfeet contributes to the weakening of the integrity of CNS vasculature and initiates their secretion of neuroinflammatory factors signaling for oligodendrocyte precursor cells (OPCs) to cluster around cerebral capillaries instead of axons (Cashion et al., 2023; Ortiz and Eroglu, 2024). Within MS lesions, astrocytes become reactive and reduce their coverage of the vasculature, resulting in perturbation of the basal lamina and disruption of the NVU (Figure 3). Additionally, astrocytes have been proposed to play a role in neurovascular coupling (NVC) and the regulation of neural activity in response to cerebral blood flow, which is diminished in MS (Stickland et al., 2019; Lia, 2023). Likewise, interactions between the NVU and ECM regulating trafficking between the CNS and blood may modulate synaptic plasticity (De Luca, 2020). The role of astrocytes in MS has been extensively reviewed, revealing robust morphological and transcriptomic changes that both contribute and respond to disease (Salles, 2022; Ortiz and Eroglu, 2024) (Figure 4). 

**Figure 5:**
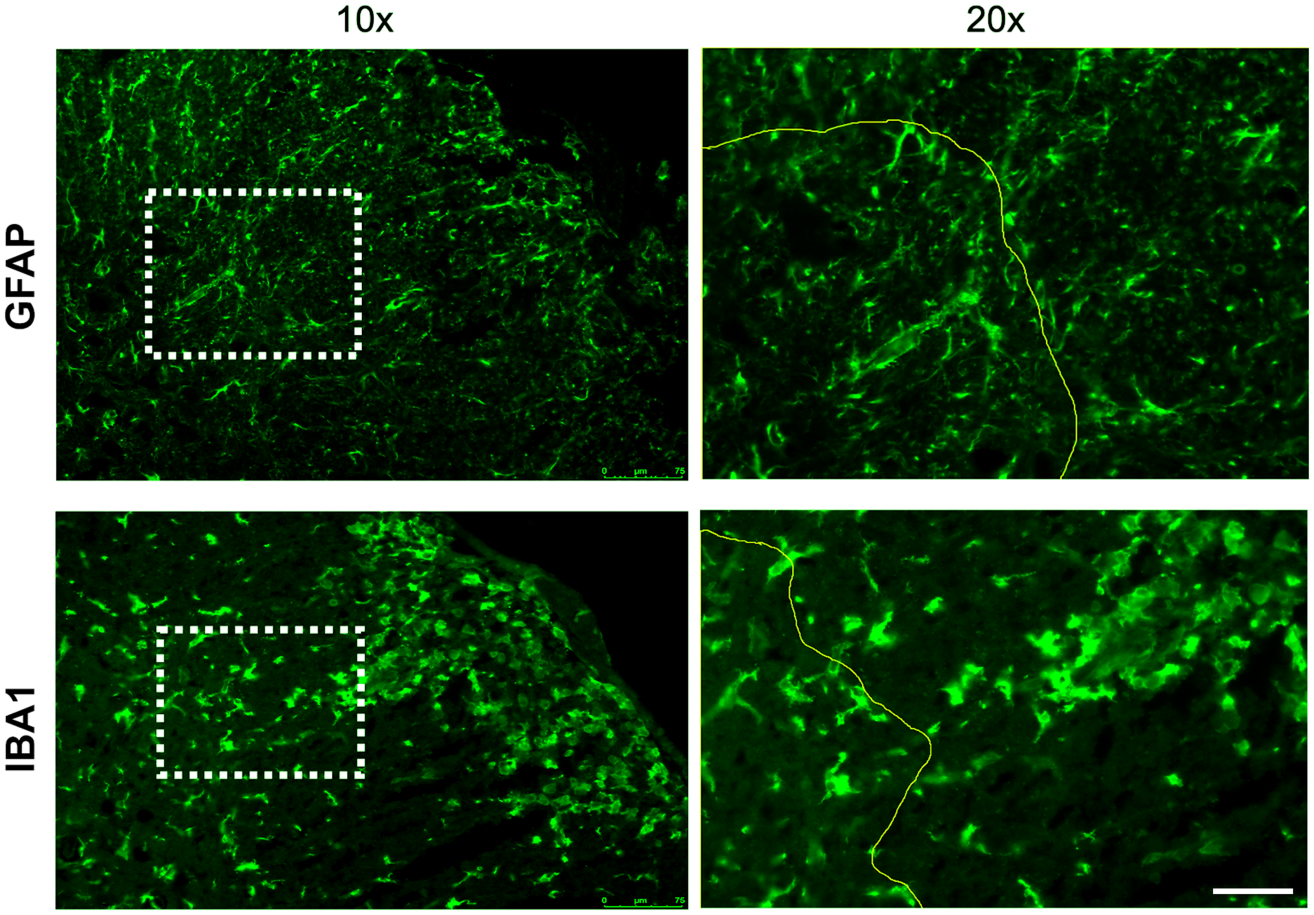
Reactive gliosis and immune cell infiltration in MS lesions. Immunohistochemistry shows morphological changes in astrocytes (GFAP+) and microglia/macrophages (Iba1+) within the lesion core (white matter, right of yellow line) compared to adjacent gray matter (left of yellow line) in an EAE mouse with clinical score 2. Scale bars 75uM.

Microglia, while not an integral part of the NVU, influence NVU integrity in a number of ways during inflammatory episodes. For example, microglia can exist in multiple states that may be either anti-inflammatory (M1) or proinflammatory (M2; Ding et al., 2021; Mirarchi et al., 2024), with the pro-inflammatory state dominating in areas of MS lesions (Figures 4, 5). Proinflammatory microglia secrete a range of cytokines including TNF-*α*, nitric oxide (NO) and matrix metalloproteinase (MMPs), and may modulate neurovascular ECM (Lloyd et al., 2019) and the expression of tight junction proteins in BECs (Smith, 2022; Smith, 2022). Due to a high level of motility, microglia quickly migrate to damaged vessels and extend their processes to regions vacated by astrocyte endfeet (Bisht et al., 2021), a response that is muted in the setting of widespread neuroinflammation (Smith, 2022) possibly because of reduced recruitment to blood vessels via decreased ATP release through PANX1-P2RY12 receptor coupling (Bisht et al., 2021). The most upregulated pathways in EAE microglia include Wnt signaling, ECM and synaptic transmission pathways, indicating the multifaceted roles of microglia during disease (Ahn, 2023).

While the majority of studies in MS and animal models such as EAE have focused on the pathology and disruption of the NVU in the spinal cord, in part because lesion burden is high and clinical motor dysfunction can be readily quantified, it is clear that MS pathology in humans also involves substantial inflammatory and neurodegenerative changes in the cortex, deep gray matter, and optic nerve. The cell and molecular mechanisms underlying lesion development in these regions are less well understood, although recent studies using EAE models and cranial window based analyses (Yousefi, 2025) suggest that cortical NVU breakdown in EAE is associated with early pericyte injury, BBB dysregulation, and neuroinflammation. These observations support the hypothesis that widespread effects on the NVU of the cortex contribute to neuroinflammation and neurodegeneration and emphasize the need to integrate a global understanding of NVU biology to obtain a complete picture of disease mechanisms.

### B cell modulation of the NVU in MS

A characteristic of MS is the influx of T and B cells into the CNS resulting in BBB breakdown and subsequent neuronal degeneration (Silva et al., 2021; Brummer et al., 2022). In MS, B cells have been detected in white matter, meninges, and CSF (Lucchinetti, 2000) and have been shown to have a higher pro-inflammatory profile compared to those in healthy individuals (Seals, 2022; Li et al., 2018). The success of B cell-targeted therapies like rituximab, ofatumumab, and ocrelizumab in treating different stages of the disease, preventing new lesion formation, and reducing relapse rates highlights the crucial role of B cells in disease progression (Hauser et al., 2008; Hauser et al., 2017; Montalban, 2017). While the target of B cell generated autoantibodies and inflammatory cytokines is generally considered to be neurons and glia, B cells also play an important role in modulating the NVU through their interactions with BECs. Within MS lesions, BECs upregulate adhesion molecules VCAM-1 and ICAM-1 at B cell infiltration sites, which correlate with the expression of B cell counter-receptors VLA-4 and LFA-1 and facilitate B cell migration into the CNS. Several studies have explored the therapeutic potential of targeting VLA-4 in B cells (Rodriguez-Mogeda et al., 2022) that suggest a reduction in B cell migration across BECs by disrupting VLA-4’s interaction with fibrinogen (Cannella and Raine, 1995).

In B-cell-dependent EAE models, VLA-4 deletion in B cells leads to decreased recruitment of proinflammatory B cells, Th17 cells, and macrophages into the CNS, resulting in a significant reduction of clinical symptoms that was independent of peripheral B and T cells (Zamvil, 2015). These results suggest that the effects are primarily due to the selective inhibition of B cell recruitment into the CNS. Conversely, in B-cell-independent EAE models, VLA-4 deficiency in B cells resulted in more severe EAE. This was linked to a significant reduction in regulatory B cells (Bregs) within the CNS, highlighting the importance of VLA-4 for Breg migration and their neuroprotective role (Lehmann-Horn et al., 2016). Additionally, recent clinical studies with natalizumab, a VLA-4 blocking therapy, have shown reduced but not completely blocked immune cell infiltration into the CNS (Kowarik et al., 2021). These findings suggest that the role of VLA-4 on B cells can be either proinflammatory or neuroprotective, depending on the context of the EAE model and the activation state of the B cells. For example, the differential effects of VLA-4 may reflect a subset-specific reliance on VLA-4–mediated trafficking, with regulatory B cells being particularly dependent on VLA-4 for CNS entry during neuroinflammation. We hypothesize that context-dependent differences in B-cell activation state and integrin usage help explain the opposing effects of VLA-4 loss observed in B-cell-dependent versus B-cell-independent EAE models.

In addition to their interactions with BECs, B cells also play a crucial role in modulating the NVU through their effects on glial cells. The presence of proinflammatory B cells alongside reactive glial cells suggests that B cells may significantly influence local gliosis. Astrocytes and microglia may exist in different anti- and pro-inflammatory states that can be influenced by B cells. For example, astrocytes cultured with EAE B cells but not controls resulted in morphological changes (Ahn, 2023) and could induce damage to oligodendrocytes. Transcriptional analysis of EAE astrocytes following B cell depletion suggested that B cell depletion did not reverse astrocytic neuroinflammatory pathways; instead, the top differentially expressed pathways were associated with interaction via NVC and focal adhesion kinase (FAK) pathways, suggesting that functional recovery following B cell depletion is driven by enhancements to the NVU rather than by the direct suppression of inflammation. These findings were further validated *in situ*, with consistent Cldn 5 expression around blood vessels, a significant reduction of albumin leakage, and decreased immune cell filtration into the spinal cord parenchyma in B cell depleted animals compared to controls (Ahn, 2023).

The role of B cells in MS is clearly complex. In a longitudinal study analyzing published single-cell sequencing data from Maggi (2023) found that B cell depletion was effective in suppressing activated microglia and preventing them from escalating their immune response. Specifically, depletion of CD20 B cells was predicted to affect microglial genes involved in iron/heme metabolism, hypoxia and antigen presentation. However, *in vivo* validation via MRI data demonstrated B cell depletion did not significantly reduce white matter lesion volume or mitigate the chronic inflammatory process over longer periods, particularly in paramagnetic rim lesions (PRLs). Additionally, in chronic active lesions (CALs), CD20 B-cells comprised a small percentage of all lymphocytes and were outnumbered by plasmablasts and activated T-cells. These findings suggest that while peripheral B-cell depletion is effective in preventing new lesion formation, it does not sufficiently resolve the activity of iron-laden microglia at the edge of chronic active lesions that are isolated behind a closed BBB in prolonged periods of disease (Maggi, 2023).

The interactions between B cells and components of the NVU—including BECs, astrocytes, and microglia—underscore the multifaceted role of B cells in modulating BBB integrity, leukocyte infiltration, neuroinflammation, and gliosis in MS. Further studies are needed to illuminate the complexity of B cell function within the CNS and open avenues for targeted therapies aimed at preserving NVU function and mitigating disease progression in MS.

### Current MS therapeutic landscape

Significant progress in the treatment of MS has been made since the introduction of interferon beta-1b (IFNβ-1b) in 1993 (Paty and Li, 1993). Since then, therapies with increasing efficacy, selectivity, and safety have been developed, broadening our understanding of the disease and increasing the window for therapeutic intervention (Montalban, 2017; Kappos et al., 2020). Nevertheless, there is no cure for the disease and many of the current strategies focus on targeting the immune system to reduce inflammatory cytokines or prevent the entry of immune cells into the CNS, thereby reducing acute attacks. However, emerging therapeutics are beginning to target vascular-mediated functions to slow or halt the progression of disease.

MS has long been known to be a T-cell associated disease due to robust T-cell infiltration into the CNS, leading to cytokine release and impairment of tight junctions along the BBB (Wagner et al., 2019). However, early clinical trials with purely T-cell based approaches were found to be ineffective (van Oosten, 1997; Weinshenker, 1991). Later strategies were developed to inhibit lymphocyte infiltration into the CNS (Polman et al., 2006). Approved by the FDA in 2004, natalizumab is a selective adhesion-molecule inhibitor that prevents binding of α_4_β_1_ and α_4_β_7_ integrins to their receptors, thereby modulating inflammatory reactions in the CNS (Polman et al., 2006). Treatment with natalizumab in RRMS patients reduced the risk of sustained progression of disability by 42% and the rate of clinical relapse by 68%, further supporting the role of lymphocyte migration and vascular dysfunction in disease progression. Subsequent *in vitro* studies showed that very high levels of ICAM-1 would be required to allow natalizumab to thwart T-cells’ attack on BECs effectively (Soldati et al., 2023). Other disease-modifying therapies (DMTs) have since been approved for relapsing and progressive disease, including cladribine, ocrelizumab, ofatumumab, siponimod, and ponesimod (Montalban, 2017; Giovannoni et al., 2010; Kappos, 2018; Hauser, 2020; Kappos, 2021). The continuous development of DMTs with different mechanisms of action and varying rates of disease control highlight the complex role of the BBB and the need to evaluate disease activity to determine the best course of treatment.

Since the success of rituximab, a monoclonal antibody targeting CD20, a significant amount of evidence suggesting the involvement of B cells rather than T cells has accumulated (Chisari et al., 2022). B-cell directed therapies such as rituximab, ocrelizumab and ofatumumab have investigated targeting B-cells by antibody-mediated depletion (Hauser et al., 2008; Hauser et al., 2017; Hauser, 2020), showing greater functional improvement, lower relapse rates, and reduced rate of lesions. Interestingly, treatment of teriflunomide, an oral DMT that inhibits pyrimidine synthesis and reduces T cell and B cell activation, resulted in higher annualized relapse rates compared to ofatumumab (Hauser, 2020). While teriflunomide significantly reduces B cell counts in patients, T cells are affected to a lesser extent (Gandoglia et al., 2017). More recently, chimeric antigen receptor T cell (CAR-T) technology, initially used for the development of therapies for hematological malignancies, has shown promise as a potential treatment for MS. In 2024, a fully human autologous CD19 CAR-T cell therapy (KYV-101) was used to treat two patients with progressive and refractory MS, resulting in acceptable safety profiles and reduced intrathecal antibodies (Fischbach et al., 2024). One hypothesis for the shortcoming of current B-cell targeted therapies in effectively preventing relapses is that monoclonal antibodies such as rituximab and ocrelizumab do not adequately cross the BBB to influence CNS B cell function. Increased knowledge about the involvement of the CNS vasculature in MS pathogenesis provides rationale for the clinical evolution of MS therapies that target the BBB (Monson, 2005). KYV-101, on the other hand, was observed in the CSF and expanded without signs of immune effector cell-associated neurotoxicity syndrome (Fischbach et al., 2024). These B cell directed therapies not only demonstrate B cell involvement in disease progression but also suggest a role for B cells that have already accumulated in the CNS.

There are currently multiple potential therapies available to treat MS. The majority of these are focused on modulating the effects of the immune system, either through altering inflammatory cytokines or preventing the entry of immune cells into the CNS. While originally thought to be a predominantly T cell mediated disease, the development of B cell depleting therapies have identified a central role for B cells in mediating disease progression.

## Ischemic Stroke

While not considered a classic neuroinflammatory disease, extensive data suggests there is a pivotal role for changes in the NVU in driving the ongoing pathogenesis in Ischemic Stroke (IS). IS is the leading cause of disability worldwide (Katan and Luft, 2018) and the majority of early studies focused on the loss of neuronal function, including mitochondrial dysfunction, excitotoxicity, and neuronal death (Dirnagl, 2012). It is thought that one of the leading causes of IS are plaques within cerebral vessels and infarcts from vessel lesions (Bailey et al., 2012), expanding the mechanisms of IS to include the NVU and its cellular components. Similar to the pathophysiology of multiple sclerosis, the involvement of the NVU in IS can both contribute to disease or repair of CNS tissue through processes involving glia, neurons, and matrix components of the NVU.

The deleterious effects of IS stem from several key pathophysiological factors involving the cerebral vasculature. During the acute phase of cerebral ischemia glutamatergic excitotoxicity, calcium overload, and oxidative stress are accompanied by BBB damage (Dohmen et al., 2005). Breakdown of the BBB results in increased permeability and downregulated expression of tight junction proteins such as claudin-5 (Stamatovic et al., 2019). Accompanied by the conversion of BBB endothelial cells toward a pro-inflammatory phenotype, an upregulation of adhesion molecules, ICAM-1 and VCAM-1, increases peripheral immune infiltration into the CNS (Supanc et al., 2011). The resulting leukocyte infiltration in the CNS and upregulation in inflammatory pathways triggers neuronal death and glial activation that may restrict recovery and repair of the tissue. Hence, it is critical to target repair of the NVU and BBB as treatment avenues for IS.

Glial cells of the NVU contribute to BBB breakdown by upregulation of proteolytic enzymes, including MMPs. MMP-9 is involved in degradation of the extracellular matrix and basal lamina, resulting in infiltration of peripheral immune cells (Ramos-Fernandez et al., 2011; Wang, 2018). While the exact role of MMP-9 in the development of IS has yet to be defined, it is clear there is a strong association of increased MMP-9 with severity of IS and worsened functional outcome (Ramos-Fernandez et al., 2011; Horstmann et al., 2003; Moldes, 2008; Montaner, 2001). Enhanced expression and activity of MMP-9 is localized around blood vessels, accompanied by increased neutrophil infiltration and macrophage activation (Rosell, 2006). Conversely, inhibition of MMP-9 activity has been shown to rescue function, including visual cortex plasticity after IS and motor outcomes following traumatic brain injury (Akol et al., 2022; Wang et al., 2000). In a recent study using *Mmp9* knockout mice and neutralizing antibodies to MMP-9, brain tissue injury and BBB breakdown were significantly attenuated compared to controls (Ji, 2023). This highlights the potential to utilize neutralizing antibodies to MMP-9 to inhibit enzymatic activities that degrade the tight junction proteins that protect the BBB. Hence, much research in the last two decades has focused on neuroprotective strategies targeting BBB function, including MMP-9 inhibition (Romanic et al., 1998). Selective inhibition of MMP-9 in animal models of IS significantly reduces infarct size perhaps by inhibiting loss of microvascular integrity and hence inflammatory response (Romanic et al., 1998). While primarily used as an antibiotic, minocycline is known for its neuroprotective effects and was found to inhibit MMPs (Guerin et al., 1992; Sapadin and Fleischmajer, 2006). Additionally, statins, a class of cholesterol-lowering medications, have been considered as neuroprotective agents after studies in IS models showed a reduction in hemorrhagic transformation and increase in time to onset of IS (Zhang et al., 2009). While promising data have demonstrated the potential for therapies targeting MMP and other pathophysiological contributors, there is a lack of data showing efficacy in patients and more research is needed.

### The multifaceted role of B cells in the NVU of ischemic stroke

In IS, BBB disruption occurs within hours, followed by secondary breakdown within 24–48 h (Okada et al., 2020). This leads to infiltration of peripheral immune cells, including B cells, into the CNS, where they contribute to both acute and chronic neuroinflammation. Elevated levels of CXCL13, a B cell-attracting chemokine occur in cortical vessels of the ischemic hemisphere within 24 h post-IS, suggesting early recruitment of B cells across the compromised NVU in IS (Rayasam et al., 2022).

B cells persist in the brain up to 10 weeks after IS and remain elevated in peripheral blood for at least 12 weeks (Weitbrecht et al., 2021; Li et al., 2021), indicating their potential role in long-term modulation of IS pathology. These cells appear to have both neuroprotective and neurotoxic effects, depending on their subset and activation state. For example, Bregs increase post-IS and are associated with improved outcomes (Li et al., 2021; Urra et al., 2009), while age-associated B cells may intensify inflammation by activating microglia or releasing mixed cytokine profiles (Malone et al., 2023).

Differences in the functional roles of B cell subsets in IS may influence the integrity of the NVU and affect therapeutic outcomes. Some studies suggest B cells influence NVU function through modulation of microglia. Specifically, IL-10-enriched B cells can reduce infarct volume, dampen pro-inflammatory cytokine expression, and promote resting microglia phenotypes (Bodhankar, 2013; Ortega, 2020). However, B-cell depletion models have shown conflicting results: some report increased infarct size (Ren et al., 2011), while others found no significant impact (Schuhmann et al., 2017). For example, IgA-producing B cells found to impair cognitive recovery contribute to vascular dementia and IS-related neurodegeneration (Zbesko et al., 2021). Additionally, IS has been shown to result in autoreactive B cell responses against neuronal antigens (Ortega et al., 2015), suggesting that B cells may contribute to long-term neuroinflammation that may secondarily affect the NVU. These discrepancies may reflect differences among B cell subsets or experimental models.

Together, these findings highlight the dualistic and context-dependent roles of B cells in NVU integrity and IS recovery. Future research should prioritize dissecting how specific B cell subsets interact with NVU components, especially microglia, astrocytes, endothelial cells, and pericytes, to influence IS pathology and repair.

## Parkinson’s disease

**Figure 6:**
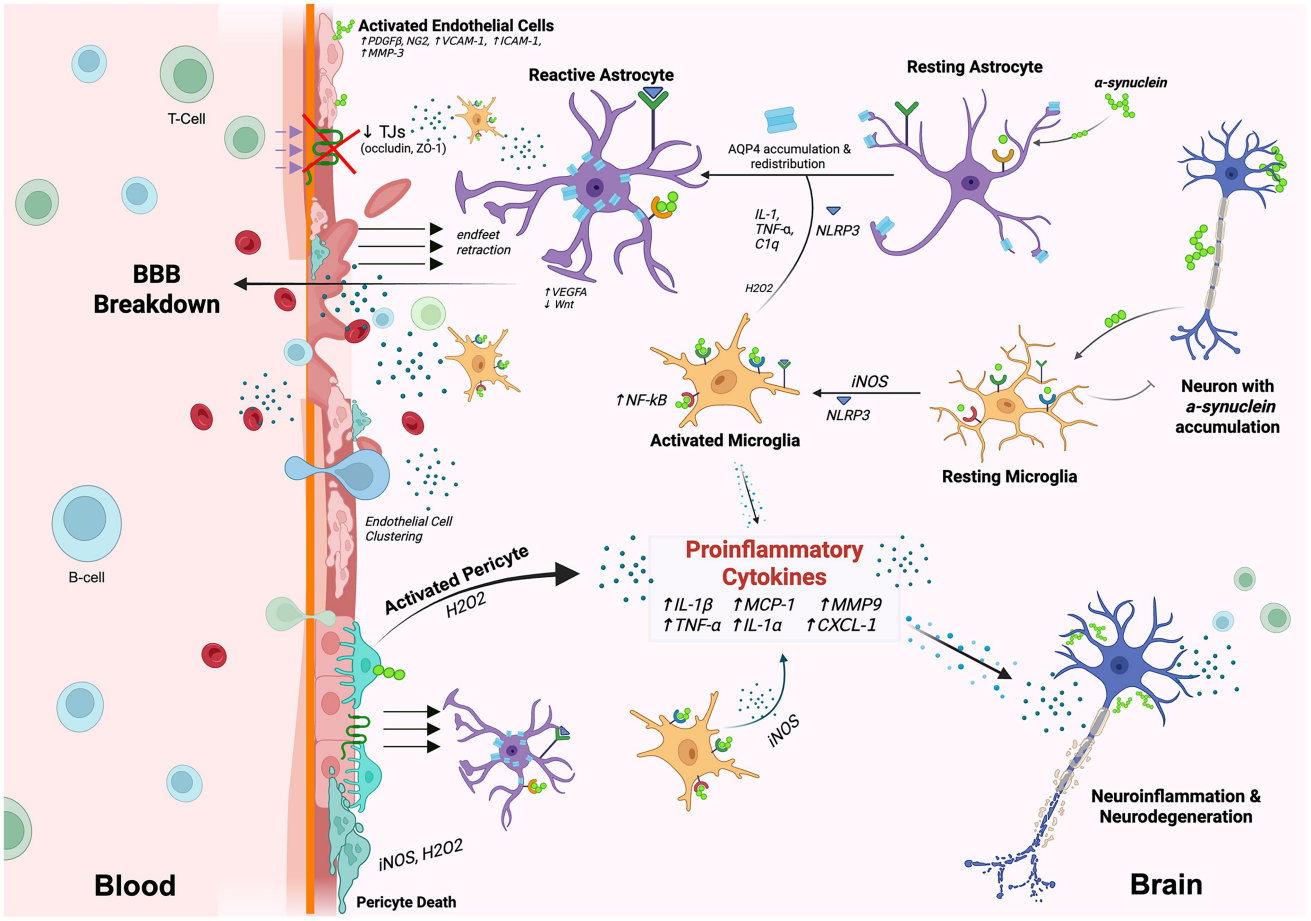
NVU dysfunction in Parkinson’s disease. In PD, misfolded α-synuclein accumulates in neurons and is transmitted to cells within the NVU. α-Synuclein induces microglial activation through interactions with TLR2, CD11b, and the NLRP3 inflammasome. Activated microglia promote astrocyte activation and pericyte dysfunction through inflammatory signaling and the release of reactive oxygen species (i.e., H_2_0_2_). Astrocytes, pericytes, and endothelial cells also internalize α-synuclein directly, driving them toward a proinflammatory state. Pericyte activation and altered vessel density contribute to microvascular instability and vascular leakage. Astrocytic dysfunction is characterized by abnormal upregulation and mislocalization of aquaporin-4 (AQP4), leading to impaired endfoot polarization and retraction from the vascular surface. Disruption of astrocytic endfeet, together with pericyte activation, compromises BBB integrity. Proinflammatory cytokines released by activated NVU cells further exacerbate dysfunction. Endothelial cells downregulate tight junction proteins such as occludin and ZO-1, promoting BBB breakdown, immune cell infiltration, and neuronal degeneration.

Parkinson’s Disease (PD) is the second most common neurodegenerative disorder and is increasing in prevalence. It is estimated that the number of individuals affected with PD will be in the order of 14 million by 2040 (Dorsey et al., 2018). Patients with PD typically present with rigidity, bradykinesia, and resting tremors due to the progressive degeneration of the nigrostriatal system, a major dopamine pathway in the brain that controls voluntary movement and balance (Paul and Elabi, 2022). Histologically, PD is characterized by the formation of Lewy bodies containing aggregations of a-synuclein (a-syn) in dopamine neurons that leads to their death. Encoded by the SCNCA gene, a-syn is abundantly expressed in the nervous system and is the most effective predictive biomarker for evaluation of PD (Stefanis, 2012). The hallmark accumulation of a-syn, formation of Lewy bodies and neurodegeneration in PD appear to be closely intertwined with neuroinflammatory signaling and microvascular alterations impacting the NVU, which in turn exacerbate disease progression via BBB disruption and sustained neuroinflammation (Figure 6).

The loss of BBB integrity is detected at early stages of disease in PD mouse models and is associated with NVU cell activation, angiogenesis, neurodegeneration and microvascular changes (Sweeney et al., 2019; Elabi et al., 2021; Paul and Elabi, 2022). This is evident with the decrease in endothelial cell tight junction proteins such as occludin and ZO-1 (Kuan, 2016). Further, the formation of pathological clusters of endothelial cells in PD brain tissue is associated with vascular degeneration and capillary fragmentation (Guan et al., 2013) along with increased brain region-specific expression of VCAM-1, ICAM-1, and MMP-3, indicative of endothelial activation (Lau, 2023). Previous studies also have found that astrocytic VEGFA increases in response to a-syn accumulation and promotes vascular permeability (Lan et al., 2022).

The a-syn aggregates that form in PD directly interact with the NVU to regulate BBB permeability and exacerbate neuroinflammation (Lan et al., 2022; Wang et al., 2023). *In vitro*, treatment of monomeric a-synuclein activated NG2 + pericytes, causing them to induce hyperpermeability in neighboring endothelial cells and release proinflammatory cytokines (Dohgu et al., 2019). These activated pericytes can also stimulate microglia by releasing IL-6 and inducing iNOS expression (Matsumoto, 2018). Consistent with these *in vitro* findings, *α*-syn transgenic mouse models exhibit early pericyte activation and dynamic changes in vessel density (Elabi et al., 2021), which may reflect compensatory angiogenesis in response to vascular leakage (Sweeney et al., 2016), supporting the concept that pericyte dysfunction contributes to microvascular instability and progressive NVU pathology in PD.

Given that the functions of pericytes and astrocytes are closely linked in the regulation of BBB integrity (Bonkowski et al., 2011), it is predictable that there are PD related changes in astrocytes. Aquaporin-4 (AQP4), the most abundant water channel in the CNS (Oshio et al., 2004; Papadopoulos et al., 2004), primarily localized to perivascular astrocytic endfeet, with higher expression at pericyte-facing membranes (Gundersen et al., 2014; Cohen-Salmon et al., 2021), is abnormally upregulated (Prydz et al., 2017) and redistributed to the soma and proximal processes in PD models (Gu, 2010). Consistent with these observations, genetic loss of AQP4 attenuates astrocyte hypertrophy following injury, supporting a role for AQP4 in regulating astrocyte reactivity (Fukuda and Badaut, 2012). Moreover, AQP4 mislocalization impairs the structural integrity of astrocytic endfeet (Warth et al., 2004), suggesting that disrupted endfoot polarization contributes to compromised BBB regulation in PD. Together, these findings highlight that alterations in both astrocytic endfeet and pericyte function may contribute to impaired BBB regulation and progressive neurovascular pathology in PD.

Activated astrocytes and microglia release proinflammatory factors that can degrade tight junction proteins in PD (Matsumoto, 2018; Gao et al., 2023). PD mouse models and recent sequencing studies demonstrate that a-synuclein can directly stimulate and activate neighboring microglia and astrocytes (de Rus Jacquet et al., 2023; Bido et al., 2021). Several pathways are involved in modulating glial activation via a-syn, including the promotion of microglial TLR2 expression (Lv, 2023; Lee et al., 2010). Importantly, the overactivation of astrocytic and microglial NLRP3 inflammasome, as seen in TBI models (Zhang et al., 2025), is a major contributor of PD pathology. A-syn directly promotes NLRP3 activation, which creates a positive feedback loop of glial cell activation, BBB disruption, a-syn accumulation and neuronal damage (Zhang et al., 2025; Candelario-Jalil et al., 2022). Targeting the NLRP3 inflammasome has been shown to promote disease prevention and neuroprotection through its inhibition in microglia (Gordon, 2018; Lee et al., 2019) or its degradation by Parkin protein (Zhang, 2023). Additionally, a 3D human BBB model using LRRK2 G2019S mutant astrocytes, showed high NLRP3 expression to be associated with impaired capillary support. Here, astrocytic inflammation and blood vessel integrity was rescued by MEK1/2 inhibition (de Rus Jacquet et al., 2023).

Impaired crosstalk between neurons and cells of the NVU may be a critical factor in PD pathology. Microglial activation can directly promote astrogliosis through the reactive oxygen species (ROS), including H₂O₂, via STAT1 and STAT3 signaling (Hou et al., 2017). Additionally, disrupted neuronal-microglial communication via the CXCL/CX3CR1 axis leads to microglial activation, leading to increased iNOS expression and neurodegeneration (Castro-Sánchez, 2018). Astrocytes, which normally support the BBB by releasing Wnt to maintain Wnt/Frizzled-1/*β*-catenin signaling in endothelial cells, have reduced Wnt expression in PD models. Inflammation-driven disruption of Wnt signaling further reduces tight junction integrity and promotes immune cell infiltration, causing a feed-forward loop accelerating NVU breakdown and disease progression (Marchetti et al., 2013).

These findings highlight the complex and critical crosstalk of NVU cells in coordinating proinflammatory microenvironments and reducing BBB integrity in PD. Ultimately, pericyte and glial cell activation along with increased endothelial cell permeability can alter the state of the NVU in PD and ultimately compromise BBB integrity, further exacerbating neuroinflammation and disease progression.

### The role of B cells and other infiltrating immune cells in modulating the NVU in PD

Disruption of the BBB in PD facilitates the infiltration of peripheral immune cells and proteins into the CNS, contributing to persistent neuroinflammation and NVU impairment. Post-mortem studies show increased infiltration of CD4 + and CD8 + T cells in PD brains, particularly in the substantia nigra, where they may directly contribute to neuronal injury (Brochard et al., 2008). Similarly, classical monocytes are elevated in PD and may enter the CNS, although whether they directly interact with the NVU needs to be further explored (Grozdanov et al., 2019; Thome, 2025).

While T cells and monocytes have received significant attention, emerging evidence implicates B cells in modulating NVU function in PD. PD patients show increased transitional and memory B cells and altered cytokine profiles (Sulzer et al., 2017). Additionally, IgG deposits have been detected in the substantia nigra and Lewy bodies (Orr, 2005), linking humoral immunity to PD pathology. These B cell populations may affect the NVU both directly and indirectly. Bregs, which are typically anti-inflammatory, are increased in PD and show conflicting roles. While some studies report neuroprotective effects, others suggest limited or even pathogenic properties (Orr, 2005; Zhang, 2023). Additionally, PD patients with cognitive decline have fewer but more activated B cells, which may amplify inflammatory responses at the NVU (Scott et al., 2023). In animal models, B cell depletion has been shown to exacerbate motor deficits and dopaminergic neuron loss, while their presence appears to mediate neurotoxic damage and support neuronal survival (Roodveldt et al., 2024). Future studies should continue to investigate the multifaceted functions of B cells in PD, particularly whether specific subsets may serve as disease-attenuating targets to preserve NVU integrity over the course of disease progression.

## Alzheimer’s disease

Dysregulation of the NVU is a critical pathophysiological element in Alzheimer’s Disease (AD), the leading cause of cognitive decline in the elderly population (Mucke, 2009; Yu et al., 2020). While the classic hallmark of disease is the presence of tau and amyloid-*β* peptide deposits in the brain, many of these deposits have been found near blood vessels and shown to contribute to changes in the cerebral vasculature (Mucke, 2009; Halliday et al., 2013; Yamazaki and Kanekiyo, 2017). Indeed, the term “vascular dementia” has been used to describe AD and dementia-related diseases for several decades (Canobbio et al., 2015). To date, patients with AD exhibit many vascular dysfunction related symptoms, including increased risk of stroke, hypertension, and diabetes (Morris et al., 2014; Tolppanen et al., 2013).

**Figure 7:**
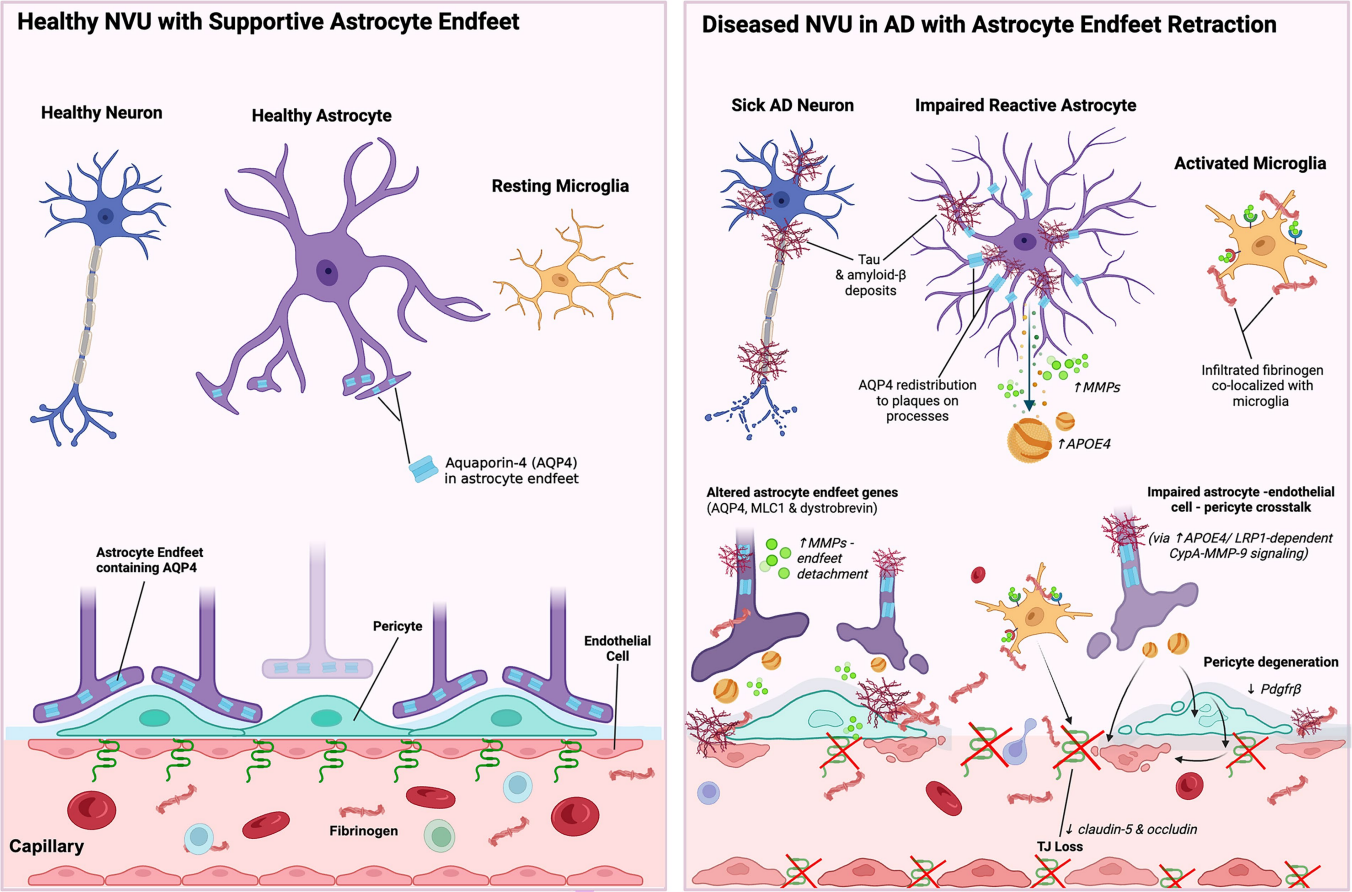
NVU dysfunction in Alzheimer’s disease. In Alzheimer’s disease, neurovascular unit dysfunction is marked by reduced tight junction proteins, leading to blood–brain barrier leakage, fibrinogen extravasation, microglial activation, and inflammation. Amyloid-ß damages astrocytic endfeet and promotes early pericyte loss, impairing neurovascular coupling and barrier integrity. Mislocalization of AQP4 and altered endfoot-associated proteins lead to endfeet detachment, increased astrocyte reactivity, and MMP release, further weakening the BBB. Pericyte degeneration, driven by reduced PDGFRß signaling and astrocytic ApoE4-mediated disruption of pericyte-endothelial crosstalk, exacerbates vascular dysfunction and permeability.

AD can manifest as a complex combination of several pathologies, many of which involve the vasculature and BBB alterations (Figure 7). A growing body of evidence demonstrates the strong association between atherosclerotic plaques in AD and the classic hallmarks of disease, including neurofibrillary tangles (Yarchoan, 2012). One study of post-mortem brains found more than 77% of AD brains exhibited atherosclerosis (Yarchoan, 2012), while others have proposed that vascular injury may precede or be an early predictor of neurodegeneration in AD (Halliday et al., 2013). While many of the studies of AD are from post-mortem brain tissues, recent studies in human subjects with genetic risk factors for AD have shown an association between BBB permeability and the *APOE4* risk factor, demonstrating the potential contribution of vascular dysfunction in AD (Halliday et al., 2013).

Early studies of post-mortem AD brains demonstrated the accumulation of albumin and IgG in near plaques surrounding blood vessels (Wisniewski et al., 1997; Wisniewski and Kozlowski, 1982), consistent with vascular dysfunction in AD. Conversely, other studies demonstrated no significant increase in serum protein extravasation in AD compared to control brains, suggesting extensive heterogeneity of AD pathology and highlighting the need for continued investigation into biomarkers and their relationship to disease severity (Alafuzoff et al., 1987; Tomimoto, 1996).

As described previously, BBB integrity is controlled by several components in the NVU. Studies of post-mortem human brains have shown significantly reduced tight junctions’ proteins, including claudin-5 and occludin, in amyloid-*β*-laden capillaries (Carrano, 2011). This was associated with microglia activation and increased fibrinogen deposition, suggesting vascular leakage and inflammation. Additional studies further demonstrate fibrinogen colocalization with microgliosis and BBB disruption, contributing to neuroinflammatory amplification (Ryu and McLarnon, 2009). One potential pathway of BBB damage is the finding that amyloid-*β* accumulation can cause dysfunction of astrocytic endfeet and pericyte loss, which together contribute to impaired neurovascular coupling (Figure 7) (Winkler et al., 2014; Diaz-Castro et al., 2023). Altered expression of astrocytic endfoot-associated genes, including AQP4, MLC1, and dystrobrevin, has been identified in postmortem and transcriptomic studies of individuals with dementia (Simon et al., 2018). Furthermore, AQP4 is upregulated in the cortex (Zeppenfeld et al., 2017; Hoshi, 2012) and redistributed from the perivascular endfeet to astrocytic processes surrounding amyloid-β plaques in both AD patients and mouse models (Hoshi, 2012; Wilcock et al., 2009; Smith et al., 2019), suggesting impaired astrocytic polarity and function (Kress et al., 2014). The persistent redistribution of AQP4 is associated with elevated astrocyte reactivity and release of MMPs, which contributes to endfoot detachment and BBB weakening in AD (Rempe et al., 2016).

Several imaging studies have demonstrated the importance of BBB integrity as a contributing factor in AD. An MRI study showed a difference in temporal pattern in subjects with AD compared to the healthy elderly group (Starr, 2009). While overall leakage as a result of BBB damage did not differ between the two groups, the difference in compartmental leakage suggests that vascular changes are an early event in AD. A later study using MRI showed an age-dependent BBB breakdown in the hippocampus, suggesting that cognitive impairment due to aging may also be a consequence of BBB injury (Montagne et al., 2015). This damage to the BBB was found to be a consequence of injury to pericytes that are critical for maintenance of the BBB and regulation of peripheral immune cell entry into the CNS. Other recent studies suggest that pericyte loss may represent an early hallmark of neurodegeneration in AD and related diseases (Shi et al., 2020; Liu et al., 2025). Substantial pericyte loss, along with reduced PDGFRβ expression, has been identified in post-mortem AD brain tissue (Butts et al., 2024). Similarly, in adult and aged pericyte-deficient Pdgfrβ+/− mice, pericyte loss disrupts tight junction integrity, leading to increased nonspecific paracellular endothelial transport and BBB impairment (Bell et al., 2023). Further, astrocyte-derived ApoE4 has been shown to promote pericyte degeneration and BBB breakdown in AD through crosstalk with pericytes and endothelial cells via aberrant LRP1-dependent CypA–MMP-9 signaling (Halliday et al., 2016; Jackson et al., 2022). Overall, accumulating evidence suggests a link between NVU dysregulation and neurodegeneration, as demonstrated by studies in patients and animal models of MS, stroke, and AD.

While imaging studies have demonstrated a strong association between vascular damage and cognitive impairment, further clinical studies are needed to assess vascular function as an early predictor or contributor to AD disease progression and severity. A greater understanding of whether BBB dysfunction is a contributing factor of disease pathogenesis, or a consequence of other pathophysiological events, is needed. Further research into the complexity of the NVU interactions in neurodegenerative disease would provide more avenues for biomarker discovery and therapeutic interventions.

## Amyotrophic lateral sclerosis

Amyotrophic lateral sclerosis (ALS) is a debilitating neurodegenerative disease characterized by motor neuron degeneration, resulting in muscle atrophy. While the initial pathology of ALS is believed to be damage to motor neurons, neurovascular alterations and impairment of the blood-spinal cord barrier (BSCB) occurs during disease.

As in MS and AD, astrocytes and microglia play vital roles in both the maintenance and function of the barrier and in the propagation of neuroinflammatory processes. In animal models of ALS, such as the SOD1^G93A^ animal, breakdown of the BSCB has been observed in the spinal cord as coinciding with an increase in microglial IL-1α, TNFα, and C1q (Lobsiger et al., 2013). Complement components such as C1q are elevated in the blood, spinal cord, and CSF of mutant SOD1 animals as well as patients with ALS (Lobsiger et al., 2013; Bahia El Idrissi et al., 2016; Mantovani et al., 2014). The induction of C1q, a vital component of the innate immune response, in motor neurons has been implicated in early disease, similar to other neurodegenerative diseases such as glaucoma and AD (Dejanovic et al., 2022; Howell et al., 2011; Stephan et al., 2012). While evidence suggests the inflammatory role of C1q in disease initiation, this is not uniform. Deletion of C1q in mutant SOD1 animals did not prevent disease initiation (Lobsiger et al., 2013) but rather resulted in a further loss of synapses. Hence, the molecular mechanisms contributing to neuronal degeneration in ALS are not fully understood.

While the exact mechanisms contributing to ALS are not fully defined, multiple preclinical and clinical studies have increased interest in targeting neuroinflammatory processes during disease progression. In a phase 2 clinical trial for NP001, an immune regulator of inflammatory macrophages, ALS patients given NP001 showed slowed symptom progression accompanied by a reduction in inflammatory biomarkers, including IL18 (Miller, 2015). Microglia/macrophages are increased in number and transition to a pro-inflammatory state during disease (Liao et al., 2012). For example, Liao et al. reported that microglia isolated from mSOD1 mice at disease onset expressed typical neuroprotective microglia markers, including CD163 and BDNF, while at end-stage disease, microglia expressed more neurotoxic markers, such as Nox2, suggesting they underwent a switch from a neuroprotective, alternatively-activated phenotype to a neurotoxic, classically-activated phenotype during disease. As microglia, together with astrocytes, are the main contributors of inflammatory processes in the CNS, it is likely that a transformation to an inflammatory phenotype results in functional barrier impairments. In fact, microglial IL1b, a pro-inflammatory cytokine, has shown to reduce BSCB function by downregulating tight junction protein expression at the barrier and increasing BSCB permeability (Liao et al., 2012). Microglia-astrocyte crosstalk also promotes astrocyte reactivity through microglial release of IL1b, further exacerbating neuroinflammation (Liao et al., 2012). Overall, studies suggest that in ALS, BSCB function is compromised in areas of motor neuron degeneration, and microglia and astrocytes may play a key role in contributing to the neuroinflammation that drives vascular leakage in the CNS.

## Conclusion

Across several neurodegenerative diseases, including MS, PD, IS, AD, and ALS, dysfunction of the NVU appears to be the common pathological feature, linking vascular, glial, and immune processes. While each disease has distinct initiating factors and affected molecular pathways, disruption of BBB integrity, reactive gliosis, and immune cell infiltration consistently drive neuroinflammation and neuronal injury. Our review highlights the central role of immune-NVU interactions, particularly the influence of B cells, glial cells, and other NVU cell populations on BBB stability and vascular signaling. The data presented here underscore how these mechanisms present in MS, with parallel pathological themes evident in PD and other neurodegenerative disorders. Collectively, these studies suggest that immune-driven glial reactivity, protease-mediated junctional disruption, disease-associated protein aggregation, and pericyte dysfunction converge to contribute to NVU breakdown and BBB failure across neurodegenerative disorders. Understanding the cellular and molecular crosstalk within the NVU provides a framework for identifying therapeutic targets that address both vascular integrity and immune modulation, offering potential strategies to slow or prevent progression across various neurodegenerative diseases.
